# Predicting childhood overweight and obesity at school entrance using healthcare, demographic, and socioeconomic data in Wales, UK

**DOI:** 10.1093/eurpub/ckag051

**Published:** 2026-04-13

**Authors:** Nida Ziauddeen, Simon D S Fraser, Sebastian Stannard, Ann Berrington, Roberta Chiovoloni, Ashley Akbari, Rhiannon K Owen, Nisreen A Alwan

**Affiliations:** School of Primary Care, Population Sciences and Medical Education, Faculty of Medicine, University of Southampton, Southampton, United Kingdom; NIHR Southampton Biomedical Research Centre, University of Southampton and University Hospital Southampton NHS Foundation Trust, Southampton, United Kingdom; School of Primary Care, Population Sciences and Medical Education, Faculty of Medicine, University of Southampton, Southampton, United Kingdom; NIHR Applied Research Collaboration Wessex, Southampton, United Kingdom; School of Primary Care, Population Sciences and Medical Education, Faculty of Medicine, University of Southampton, Southampton, United Kingdom; NIHR Applied Research Collaboration Wessex, Southampton, United Kingdom; School of Economic, Social and Political Sciences, University of Southampton, Southampton, United Kingdom; Population Data Science, Swansea University Medical School, Faculty of Medicine, Health & Life Science, Swansea University, Swansea, United Kingdom; Population Data Science, Swansea University Medical School, Faculty of Medicine, Health & Life Science, Swansea University, Swansea, United Kingdom; Population Data Science, Swansea University Medical School, Faculty of Medicine, Health & Life Science, Swansea University, Swansea, United Kingdom; School of Primary Care, Population Sciences and Medical Education, Faculty of Medicine, University of Southampton, Southampton, United Kingdom; NIHR Southampton Biomedical Research Centre, University of Southampton and University Hospital Southampton NHS Foundation Trust, Southampton, United Kingdom; NIHR Applied Research Collaboration Wessex, Southampton, United Kingdom

## Abstract

In Wales, 24.8% of children aged 4–5 years live with overweight/obesity. Obesity is linked to developing multiple long-term conditions. We aimed to predict childhood obesity using healthcare and wider demographic, socioeconomic, and area-level data. The Secure Anonymized Information Linkage (SAIL) Databank in Wales contains routinely collected individual-level anonymized data from health records and administrative data. Two subsamples were created. The first restricted to singleton births between 15 March 2010 and 28 March 2012 to include Census 2011 data. The second included births after 1 January 2014 to include early-life measurements. Age- and sex-adjusted body mass index (BMI) at 4–5 years was used to define outcome of overweight/obesity (≥91st centile). Backward stepwise logistic regression models with multivariable fractional polynomials were used to develop models in stages. Data were available on 53 815 children at 4–5 years in census and 60 990 children in early-life subsample. Maternal BMI, smoking, marital status, birthweight, ethnic group, gender, and breastfeeding at birth were retained in all models. Additional variables were retained on adding census and area-level factors but increase in discrimination (Area Under the Curve, AUC) was marginal (0.66–0.67). In the second subsample, AUC improved from 0.67 to 0.79 as factors up to weight at 27 months were incorporated. Factors from healthcare records were largely consistent with existing literature. Additional insights were provided by including census data, though increase in model discrimination was marginal. Childhood obesity can act as a mediator on the pathway to multiple long-term conditions, and risk identification tools may target early prevention.

## Introduction

Childhood overweight/obesity prevalence globally increased from 8% in 1990 to 20% in 2022 [1] and the rate of increase has been higher than that of adult obesity [2]. Overweight/obesity in childhood increases the risk of adult obesity, which increases the risk of developing long-term conditions such as cardiovascular disease and diabetes [3]. Data from five UK birth cohorts between 1946 and 2001 showed a trend towards earlier onset of obesity in more recent cohorts with two to three times greater estimated probability of overweight/obesity in cohorts born after the 1980s compared to before [4]. In the 2022/2023 academic year, the prevalence of childhood overweight/obesity at the start of primary school was 24.8% in Wales [5] and 21.4% in England [6]. Children living in more deprived areas are twice as likely to have obesity than those living in less deprived areas [5, 6]. Low socioeconomic position was associated with higher weight in the 2001 UK Millennium birth cohort, with inequalities widening from childhood to adolescence [7].

Analysis of data from 6066 children from a UK birth cohort found that 75% of children with obesity at 7 years remained with obesity at 11 years and 16% of those with overweight at 7 years developed obesity and 20% returned to healthy weight at 11 years [8]. A longitudinal study of 5863 pre-adolescent children found that those of a healthy weight remained a healthy weight during adolescence but few children with overweight/obesity reduced to healthy weight with little evidence of new cases of overweight/obesity emerging during adolescence [9]. A meta-regression of 48 studies found a high degree of tracking of body mass index (BMI) over time regardless of age of BMI measurement but was strongest in the pubertal period (10–14 years) and adulthood [10]. Adults with obesity were at higher risk of developing two or more long-term conditions (five times higher for two and 12 times higher for four or more conditions) and developed conditions earlier than adults of healthy weight [11]. Globally in 2015, high BMI contributed to 4.9% of disability-adjusted life years from any cause with over a third of high BMI related disability-adjusted life years in people with BMI <30 kg/m^2^ [2]. However, decrease in adiposity into adulthood has been shown to be associated with reduced and similar risks to those with consistently normal BMI through childhood [3, 12].

There is currently no early identification system during pregnancy or early-life to detect those at high risk of childhood obesity. As part of the Studying Lifecourse Obesity PrEdictors (SLOPE) study, we utilized anonymized routinely collected antenatal and birth records linked to child health records for births in Hampshire, England, between 2003 and 2013 to develop prediction models for the risk of childhood overweight/obesity. Models were developed at stages corresponding to statutory healthcare visits for women and children in England (first antenatal booking appointment, birth, child aged around 1 year and around 2 years) [13]. Models were internally and externally validated [13, 14]. Maternal factors from pregnancy included in the model remained consistent across the stages but model performance improved across the stages from 0.66 at booking appointment to 0.83 at child age around 2 years. Stakeholders at a workshop conducted as part of this project suggested that family-focused interventions were important to prioritize in early childhood as this may provide an opportunity to change habits and trajectories before these become more established [15]. The transition to primary school is an important time with positive experiences during this time linked to improved social, emotional, and educational outcomes [16] which in turn was linked to subsequent mental health and weight in adolescence [17].

In this study, we aimed to explore how well childhood obesity at school entrance age can be predicted using healthcare and wider demographic, socioeconomic and area-level data using linked health and administrative data in another UK country: Wales. We also aimed to map the predictor variables across pre-conceptualized early-life domains [18].

## Methods

This analysis is part of the Multidisciplinary Ecosystem to study Lifecourse Determinants and Prevention of Early-onset Burdensome Multimorbidity (MELD-B) project [19] examining the role of early-life factors as childhood obesity can act as a mediator on the pathway to multiple long-term conditions [20]. As part of MELD-B, we previously conceptually identified 12 domains of early life factors which may be important for multimorbidity risk [18], and we explored how these conceptualized domains are represented in the variables considered for prediction.

### SAIL databank

The Secure Anonymized Information Linkage (SAIL) Databank (www.saildatabank) holds anonymized, routinely collected individual-level data for all Welsh residents using National Health Service (NHS) Wales. Each individual is assigned a unique identifier called an anonymized linking field (ALF) to ensure anonymity and confidentiality while enabling individual level data linkage across different datasources.

We used the SAIL MELD-B children and Young adults e-cohort (SMYC). Details of the datasources used are described in detail elsewhere [21]. The SMYC cohort was restricted to individuals born between 1 January 2000 and 31 December 2022 with both residency and health data available before 18 years of age. Individuals had to be linked to a consistent maternal record (linked to at most one mother) to be included in SMYC. This led to a sample of 896 155 individuals.

For this analysis, we further restricted to those with an outcome measurement in the National Community Child Health (NCCH) data between 4 and 5 years (measurements from the Child Measurement Programme) and with a mother identifier as we intended to use maternal variables.

### Subsamples

We created two subsamples. The first was restricted to singleton births between 15 March 2010 and 28 March 2012 to enable inclusion of administrative data from the 2011 Census [Office for National Statistics (ONS) 2011 Census Wales]. This was a year before and after the 2011 Census date and allowed for analysis of additional factors only available in the Census. Early-life data were not available for this period.

The second subsample was restricted to singleton births between 2014 and 2018 to enable inclusion of child weight measurements from early-life recorded as part of statutory health checks around 6, 15, and 27 months. The sample was restricted to births up to 2018 to allow inclusion of information at 4–5 years.

### Outcome

The Child Measurement Programme (CMP) for Wales measures the height and weight of children in Reception (primary school entry, age 4–5 years). Measurements are carried out by school nursing teams.

BMI was calculated as weight/height^2^ and converted to age- and sex-adjusted BMI z-scores according to UK 1990 growth reference charts [22]. The outcome of childhood overweight/obesity was specified using the cut-off of 91st percentile (*z*-score +1.33). This cut-off is used for national guidance on clinical management of childhood overweight in the UK [23, 24].

### Variables considered for inclusion in the prediction model

#### Maternal factors during pregnancy from healthcare records

Maternal age (in years) at pregnancy was recorded. Maternal BMI was calculated using weight and height, available at the first antenatal booking appointment in the Maternity Indicators Dataset (MIDS). For mothers with missing data on height and/or weight or no record in MIDS, we linked to adult BMI recorded in other datasources using the harmonization methodology developed at Swansea University [25]. Maternal BMI records between 1 year before conception to child’s date of birth were extracted. If more than one weight/height record was available during this period, a priority order based on the timing of measurement was used: conception to 12 weeks, 0–3 months preconception, 3–6 months preconception, 6–9 months preconception, 9–12 months preconception, and 12–24 weeks of pregnancy.

Smoking during pregnancy was recorded in maternity and child health data sources as non-smoker, gave up during pregnancy, smoker and smoker in household. Records of mothers with missing data on smoking during pregnancy were linked to GP records between one year before conception and child’s date of birth. Detailed codes were used to record smoking which was condensed to non-smoker, current smoker, ex-smoker, and stopped smoking. The priority order for repeat smoking records was: conception to 12 weeks, 12 weeks of pregnancy-birth, 0–3 months preconception, 3–6 months preconception, 6–9 months preconception, and 9–12 months preconception. Smoking status was condensed to smoker and non-smoker due to differences in recording between the different sources.

Parity was categorized as 0, 1, 2, and 3 or more. Marriage indicator at birth registration was used to derive marital status as married or living with partner, not married, and partner at different address, and single parent. Marital status from the Census was used if marriage indicator at birth registration was missing.

We linked to maternal GP records to identify the following pre-existing diagnoses: anaemia, asthma, coronary heart disease, type 1 and type 2 diabetes, endometriosis, epilepsy, hyperthyroidism, hypothyroidism, irritable bowel disease, learning disability, polycystic ovarian syndrome, venous thromboembolism, and mental health conditions. These conditions were identified in a review of guidelines, recommendations and policy reports as important indicators of preconception health [26]. Depression, anxiety, stress, and anaemia were restricted to diagnosis within 5 years preconception as deemed more relevant to the pregnancy and due to the possibility of experiencing these for a shorter period. Maternal mental health conditions was categorized as anxiety, depression or stress and severe mental health conditions (bi-polar, schizophrenia, psychotic depression, puerperal psychosis, psychosis).

#### Census

Maternal educational attainment was categorized as no qualifications, O levels or equivalent, A levels or equivalent, degree/higher or equivalent and foreign qualifications. Parents country of birth, number of cars in household, unpaid carer in household, parent disability affecting activities, parent health, number of people per bedroom and main household language were self-reported.

#### Birth (from healthcare and administrative records)

Birthweight, gestational age at birth and mode of birth were recorded at birth. Breastfeeding was recorded at birth, 7 days, 10 days, 6–8 weeks, and 6 months, and age in weeks when breastfeeding stopped. This was condensed to breastfeeding at birth (point with most data recorded). Ethnic group was recorded as White, Asian, Mixed, Black, and Other. Welsh Index of Multiple Deprivation (WIMD) for the child was linked using the closest record to birth up to a maximum of 6 months after birth. Urban/rural area of mother’s residence was from the birth registration record.

#### Early-life (from healthcare records)

Breastfeeding recorded at 6–8 weeks and 6 months and age in weeks when breastfeeding stopped was used to derive breastfeeding at 6–8 weeks. Child weight was recorded at 6, 15, and 27 months as part of statutory health visits. Weights recorded within 2 months before or after the statutory visit age were used to calculate weight, e.g. weight recorded between 4 and 8 months was classified as the 6 month visit weight if only one measurement was recorded during this period.

### Statistical analysis

All analysis were performed using Stata [27]. We adjusted for clustering by mother by including cluster-robust standard errors as some women had more than one pregnancy in the dataset. The variables with more than 5% missingness in the census subsample were maternal BMI (73.5%), maternal smoking (23.6%), maternal mental and physical health conditions (33.2%), mode of birth (29.7%), and parity (16.4%). Similarly in the early-life subsample, these were early-life weight (71.3%–84.9% depending on age at measurement), maternal BMI (37.5%), pre-existing physical health condition (31.8%), mental health condition (23.2%), breastfeeding at 8 weeks (19.0%), mode of birth (16.5%), maternal smoking (12.9%), and parity (9.1%). Multiple imputation by chained equations (MICE) was carried out using truncated regression for continuous variable and predictive mean matching for categorical variables. Missing predictor values were imputed in the sample with outcome of interest. We carried out 75 imputations of the sample generating 75 imputed datasets based on the percentage of missing data in the sample [28].

Stepwise backward elimination was used to select the variables to be included in the model [29]. Variables are removed sequentially from the model if *P* values for a variable exceeds the significance level specified at .157 (equivalent to the Akaike information criteria) [30] to reduce the risk of overfitting. Models were developed using logistic regression with overweight/obesity fitted as a binary outcome. Non-linear relationships between continuous candidate predictors and outcome were investigated using fractional polynomials. Events per variable was used to ensure the sample size was sufficient (based on a rule of thumb of 20 events per variable) [31, 32].

Models were developed in stages. For the census subsample, first including factors in healthcare records and then factors in the census and healthcare records, both incorporating data available at birth. For the early-life subsample, first incorporating data collected at birth, then child aged 6 months, aged 15 months, and aged 27 months.

### Model performance

Model performance was assessed using discrimination (measure of how well the model differentiates between individuals) and calibration (agreement of predicted outcome of the model with the observed outcome on average). The area under (receiver operating characteristic) curve (AUC) was used to summarize the overall discriminatory ability of the models. The AUC was classified as: 0.6–0.7 poor, 0.7–0.8 fair, 0.8–0.9 good, and 0.9–1.0 excellent. The calibration slope was calculated for all models.

Heuristic shrinkage factors were calculated for each model [33] and the regression coefficients from the models were multiplied by the shrinkage factor to adjust for optimism.

### Ethics approval

Ethics approval for the MELD-B project was granted by the University of Southampton Faculty of Medicine Ethics committee (ERGO 66810). Approval for the use of anonymized data in this study, provisioned within the SAIL Databank, was granted by an independent Information Governance Review Panel (IGRP) under project 1377.

## Results

Supplementary Figure S1 shows the eligible sample. 12.4% of 53 815 children in the census subsample and 13.6% of 60 990 children in the early-life subsample had BMI ≥91st centile at 4–5 years. Baseline characteristics for the samples are summarized in Table 1 and were similar for both subsamples. Mean maternal age at pregnancy was 28.5 years (SD 5.9) and mean maternal BMI was 26.3 kg/m^2^ (SD 6.2) in the census subsample. Over a fifth (23% in the census subsample, 20.2% in the early-life subsample) of mothers were categorized as smokers. Over half (57.4% in census subsample, 55.4% in early-life subsample) of mothers reported breastfeeding at birth.

**Table 1.** Summary of factors and outcome for the sample using the multiple imputed data

Variable	Census subsample	Early-life subsample
	Mean ± SD	Mean ± SD
*N*	53 815	60 990
Maternal age at pregnancy, years	28.5 ± 5.9	28.9 ± 5.7
Maternal BMI, kg/m^2^	26.3 ± 6.2	26.9 ± 8.0
Birthweight, kg	3.4 ± 0.6	3.4 ± 0.6
Gestational age at birth, weeks	39.4 ± 1.8	39.3 ± 1.8
Child age at ∼6 months, kg	–	7.8 ± 2.4
Child age at ∼15 months, kg	–	10.8 ± 2.2
Child age at ∼27 months, kg	–	13.6 ± 3.8
	% (95% CI)	% (95% CI)
Maternal smoking		
Non-smoker	77.0 (76.6–77.4)	79.8 (79.5–80.1)
Smoker	23.0 (22.6–23.4)	20.2 (19.9–20.5)
Parity		
0	50.5 (50.1–51.0)	22.5 (22.1–22.8)
1	31.0 (30.5–31.4)	35.7 (35.3–36.1)
2	12.1 (11.8–12.4)	25.7 (25.4–26.1)
3 or more	6.4 (6.2–6.6)	16.1 (15.8–16.4)
Ethnic group		
White	90.9 (90.6–91.1)	89.6 (89.3–89.8)
Asian	5.2 (5.0–5.4)	5.7 (5.5–5.9)
Mixed	2.7 (2.6–2.9)	3.3 (3.1–3.4)
Black	0.6 (0.5–0.6)	0.7 (0.6–0.8)
Other	0.6 (0.6–0.7)	0.8 (0.8–0.9)
Marital status		
Married/living with partner	81.8 (81.5–82.1)	81.2 (80.9–81.5)
Not married and partner at different address	12.7 (12.4–13.0)	13.3 (13.0–13.6)
Single parent	5.5 (5.3–5.7)	5.5 (5.3–5.7)
Maternal educational attainment		
No qualifications	11.8 (11.5–12.0)	–
O levels or equivalent	32.4 (32.0–32.8)	–
A levels or equivalent	12.8 (12.5–13.1)	–
Degree/higher or equivalent	39.3 (38.8–39.7)	–
Foreign qualifications	3.8 (3.7–4.0)	–
Maternal mental health condition		
No	57.7 (57.2–58.2)	54.7 (54.3–55.2)
Anxiety, depression, stress	40.6 (40.1–41.1)	33.0 (32.6–33.4)
Severe mental health condition	1.7 (1.6–1.8)	12.2 (12.0–12.6)
Maternal pre-existing conditions		
Anaemia	5.6 (5.4–5.9)	7.0 (6.8–7.2)
Asthma	35.4 (34.9–35.9)	38.5 (38.0–38.9)
Coronary heart disease	14.9 (14.5–15.3)	10.8 (10.6–11.1)
T1 diabetes	1.1 (1.0–1.2)	1.2 (1.1–1.3)
T2 diabetes	1.2 (1.1–1.3)	1.2 (1.1–1.3)
Endometriosis	2.5 (2.4–2.7)	2.4 (2.3–2.6)
Epilepsy	2.3 (2.2–2.5)	2.3 (2.2–2.5)
Hyperthyroidism	1.7 (1.6–1.8)	1.8 (1.7–2.0)
Hypothyroidism	5.5 (5.2–5.7)	5.6 (5.4–5.9)
Irritable bowel disease	1.2 (1.1–1.4)	1.4 (1.3–1.5)
Learning disability	0.6 (0.6–0.7)	1.0 (0.9–1.0)
Polycystic ovarian syndrome	5.6 (5.4–5.9)	6.0 (5.7–6.2)
Venous thromboembolism	1.6 (1.5–1.8)	1.6 (1.5–1.7)
Mode of birth		
Vaginal	75.6 (75.1–76.0)	74.8 (74.4–75.2)
Caesarean section	24.4 (24–24.9)	25.2 (24.8–25.6)
Child sex		
Male	51.2 (50.8–51.6)	50.9 (50.5–51.3)
Female	48.8 (48.4–49.2)	49.1 (48.7–49.5)
Breastfeeding at birth		
No	42.6 (42.2–43)	44.6 (44.2–45.0)
Yes	57.4 (57–57.8)	55.4 (55.0–55.8)
Breastfeeding at 8 weeks		
No	67.3 (66.9–67.8)	56.5 (56.1–56.9)
Yes	32.7 (32.2–33.1)	43.5 (43.1–43.9)
Welsh index of multiple deprivation		
Least deprived	16.0 (15.6–16.3)	16.1 (15.8–16.4)
2	17.3 (16.9–17.6)	16.9 (16.6–17.2)
3	20.2 (19.9–20.6)	20.4 (20.1–20.7)
4	21.6 (21.2–21.9)	21.0 (20.7–21.4)
Most deprived	25.0 (24.7–25.4)	25.6 (25.2–25.9)
Urban/rural area of residence		
Urban	68.7 (68.3–69.1)	69.6 (69.2–70.0)
Town and fringe	16.1 (15.8–16.5)	15.3 (15.0–15.6)
Village	10.1 (9.9–10.4)	10.0 (9.8–10.3)
Hamlet and isolated dwellings	5.0 (4.8–5.2)	5.1 (4.9–5.3)
Parents country of birth		
Born within the UK	92.2 (92.0–92.5)	–
Born outside the UK	7.8 (7.5–8)	–
Number of cars in household		
None	18.4 (18.1–18.8)	–
1	36.0 (35.6–36.4)	–
2	38.3 (37.9–38.8)	–
3 or more	7.2 (7.0–7.4)	–
Unpaid carer		
No unpaid carer	87.0 (86.7–87.3)	–
One unpaid carer: working part-time	2.2 (2.1–2.4)	–
One unpaid carer: working full-time	3.2 (3.0–3.3)	–
One unpaid carer: unemployed or student	4.1 (3.9–4.3)	–
Two or more unpaid carers	3.5 (3.4–3.7)	–
Parent disability		
Day-to-day activities	1.9 (1.7–2.0)	–
Day-to-day activities limited a little	3.9 (3.8–4.1)	–
Day-to-day activities not limited	94.2 (94.0–94.4)	–
Parent health		
Very good health	57.6 (57.2–58.0)	–
Good health	35.4 (35.0–35.8)	–
Fair health	5.7 (5.5–5.9)	–
Bad health	1.1 (1.0–1.2)	–
Very bad health	0.2 (0.1–0.2)	–
Number of people per bedroom		
Upto 0.5 persons per bedroom	4.1 (3.9–4.3)	–
Over 0.5 and upto 1.0 persons per bedroom	45.9 (45.5–46.4)	–
Over 1.0 and upto 1.5 persons per bedroom	30.1 (29.7–30.5)	–
Over 1.5 persons per bedroom	19.9 (19.5–20.2)	–
Main language		
Main language is English or Welsh	95.2 (95.0–95.4)	–
Main language is not English or Welsh: can speak English or Welsh well or very well	3.5 (3.4–3.7)	–
Main language is not English or Welsh: cannot speak English or Welsh well or at all	1.3 (1.2–1.4)	–
Childhood overweight or obesity (≥91st centile)	12.4 (12.2–12.7)	13.6 (13.4–13.9)

Factors retained in the healthcare factors model using the census subsample included maternal variables: age, BMI, smoking, parity, ethnic group, marital status, anaemia, venous thromboembolism, and child variables: birthweight, gestational age at birth, gender and breastfeeding at birth (Table 2). Additional variables retained in the healthcare factors and census model included: unpaid carer, maternal education attainment, mother’s type of area of residence (urban/rural) and WIMD of child’s residence.

**Table 2.** Intercept and regression coefficients of the prediction models for overweight and obesity (91st centile) in children aged 4–5 years in the census subsample using multiple imputed data (*n* = 53 815)

Predictors	Healthcare records only			Healthcare records and census		
	Coefficient	95% CI	*P*	Coefficient	95% CI	*P*
Constant	−2.80	−3.51 – −2.10		−3.21	−3.91 – −2.51	
Maternal age	−0.01	−0.01 – −0.003	.001	–	–	–
Maternal BMI	0.07	0.06–0.08	<.001	0.07	0.06–0.08	<.001
Maternal smoking						
Non-smoker	Ref			Ref		
Smoker	0.34	0.27–0.41	<.001	0.32	0.25–0.39	<.001
Parity						
0	Ref			Ref		
1	−0.11	−0.18 – −0.04	.002	−0.12	−0.19 – −0.06	<.001
2	−0.06	−0.15–0.03	.22	−0.10	−0.19 – −0.01	.03
3 or more	−0.12	−0.24 – −0.002	.05	−0.20	−0.32 – −0.08	<.001
Ethnic group						
White	Ref					
Asian	0.11	−0.01–0.23	.08	0.09	−0.03–0.22	.13
Mixed	0.08	−0.09–0.24	.37	0.08	−0.08–0.25	.32
Black	0.49	0.18–0.81	.002	0.47	0.15–0.79	<.001
Other	0.32	−0.02–0.65	.06	0.31	−0.02–0.65	.07
Marital status						
Married/living with partner	Ref			Ref		
Not married and partner resident at different address	0.18	0.10–0.26	<.001	0.16	0.08–0.24	<.001
Single parent	0.20	0.08–0.31	.001	0.18	0.06–0.29	<.001
Anaemia						
No	Ref			Ref		
Yes	0.12	−0.01–0.24	.08	0.11	−0.02–0.24	.10
Venous thromboembolism						
No	Ref			Ref		
Yes	0.24	0.02–0.46	.03	0.24	0.02–0.46	.03
Birthweight	0.62	0.56–0.69	<.001	0.63	0.57–0.70	<.001
Gestational age at birth (weeks)	−0.08	−0.10 – −0.06	<.001	−0.08	−0.10 – −0.06	<.001
Gender						
Male	Ref			Ref		
Female	0.14	0.08–0.19	<.001	0.14	0.08–0.19	<.001
Breastfeeding at birth						
No	Ref			Ref		
Yes	−0.14	−0.20 – −0.08	<.001	−0.12	−0.18 – −0.05	<.001
Unpaid carer						
No unpaid carers	–	–	–	Ref		
One unpaid carer: working part-time	–	–	–	0.17	0.01–0.34	.04
One unpaid carer: working full-time	–	–	–	0.10	−0.05–0.25	.21
One unpaid carer: unemployed or student	–	–	–	0.05	−0.08–0.17	.48
Two or more unpaid carers	–	–	–	0.13	−0.01–0.26	.08
Maternal educational attainment						
No qualifications	–	–	–	Ref		
O levels or equivalent	–	–	–	0.02	−0.07–0.11	.63
A levels or equivalent	–	–	–	0.002	−0.11–0.11	.98
Degree/higher or equivalent	–	–	–	−0.11	−0.21 – −0.02	.02
Foreign qualifications	–	–	–	−0.07	−0.24–0.10	.43
WIMD						
Least deprived				Ref		
2	–	–	–	0.16	0.06–0.26	<.001
3	–	–	–	0.19	0.09–0.29	<.001
4	–	–	–	0.20	0.10–0.30	<.001
Most deprived	–	–	–	0.23	0.14–0.33	<.001
Urban/rural area of residence						
Urban	–	–	–	Ref		
Town and fringe	–	–	–	0.11	0.04–0.19	.002
Village	–	–	–	0.07	−0.02–0.17	.15
Hamlet and isolated dwellings	–	–	–	0.02	−0.12–0.15	.80
Area under the curve	0.66	0.65–0.67		0.67	0.66–0.68	
Calibration slope (standard error)	0.97 (0.02)			0.97 (0.02)		
Shrinkage factor	0.988			0.981		
Shrunken coefficient	−2.79	−3.51 – −2.10		−3.18	−3.21 – −3.16	

Only predictors significant at *P* < .157 were included in the models. Categorical variables with at least one significant category have been included.

Factors retained in the models using the early-life subsample were similar (Table 3). The same variables were retained in both census subsample using healthcare factors only and the early-life subsample at birth with the exception of mother’s pre-existing conditions. The census subsample included anaemia and venous thromboembolism whereas the early-life subsample model included coronary heart disease and hypothyroidism (at birth only) and polycystic ovarian syndrome (at birth and early-life). Child weight during early-life was retained in the early- life models.

**Table 3.** Intercept and regression coefficients of the prediction models for overweight and obesity (91st centile) in children aged 4–5 years in the early-life subsample using multiple imputed data (*n* = 60 990)

Predictors	Birth			Child aged 6 months			Child aged 15 months			Child aged 27 months		
	Coefficient	95% CI	*P*	Coefficient	95% CI	*P*	Coefficient	95% CI	*P*	Coefficient	95% CI	*P*
Constant	−9.05	−10.17 – −7.94		−10.24	−10.76 – −9.71		−15.03	−15.6 – −14.45		−14.50	−15.26 – −13.73	
Maternal age	−0.01	−0.01 – −0.01	<.001	−0.01	−0.01 – −0.004	<.001	–	–	–	−0.01	−0.01 – −0.001	.02
Maternal BMI (transformed 1)	14.45	12.41–16.49	<.001	15.78	13.52–18.04	<.001	15.51	13.18–17.84	<.001	15.59	13.16–18.03	<.001
Maternal BMI (transformed 2)	−7.03	−9.15 – −4.91	<.001	−13.96	−17.35 – −10.56	<.001	−13.55	−17.05 – −10.04	<.001	−13.92	−17.59 – −10.25	<.001
Maternal smoking												
Non-smoker	Ref			Ref			Ref			Ref		
Smoker	0.36	0.29–0.43	<.001	0.29	0.22–0.36	<.001	0.31	0.23–0.38	<.001	0.33	0.26–0.41	<.001
Parity												
0	Ref			–	–	–	–	–	–	–	–	–
1	0.03	−0.04–0.10	.35	–	–	–	–	–	–	–	–	–
2	−0.07	−0.14–0.01	.08	–	–	–	–	–	–	–	–	–
3 or more	−0.02	−0.11–0.06	.61	–	–	–	–	–	–	–	–	–
Ethnic group												
White	Ref			Ref			Ref			Ref		
Asian	0.13	0.03–0.24	.01	0.16	0.05–0.26	.01	0.25	0.14–0.37	<.001	0.24	0.10–0.38	<.001
Mixed	−0.05	−0.19–0.09	.48	−0.13	−0.28–0.03	.12	−0.17	−0.33 – −0.01	.05	−0.08	−0.27–0.11	.40
Black	0.11	−0.19–0.41	.47	−0.12	−0.43–0.2	.53	−0.21	−0.55–0.13	.27	−0.24	−0.63–0.14	.22
Other	0.14	−0.13–0.41	.32	0.12	−0.17–0.4	.39	0.18	−0.13–0.48	.24	0.05	−0.30–0.39	.80
Marital status												
Married/living with partner	Ref			Ref			Ref			Ref		
Not married and partner resident at different address	0.19	0.12–0.26	<.001	0.16	0.09–0.24	<.001	0.16	0.08–0.23	<.001	0.18	0.10–0.26	<.001
Single parent	0.16	0.06–0.26	<.001	0.13	0.02–0.24	.02	0.13	0.02–0.25	.02	0.14	0.03–0.26	.02
Pre-existing health conditions												
No	Ref			Ref			Ref			Ref		
Coronary heart disease	−0.08	−0.17–0.02	.11	–	–	–	–	–	–	–	–	–
Hypothyroidism	−0.10	−0.23–0.03	.14	–	–	–	–	–	–	–	–	–
Polycystic ovary disease	0.10	−0.02–0.21	.09	0.17	0.05–0.29	.01	0.18	0.05–0.30	.01	0.17	0.05–0.29	.01
Mental health condition												
Anxiety, depression, stress	–	–	–	0.08	0.02–0.14	.01	0.08	0.02–0.14	.01	0.06	0.00–0.13	.05
Severe	–	–	–	0.08	−0.01–0.17	.09	0.05	−0.05–0.14	.34	0.05	−0.05–0.14	.33
Mode of birth												
Vaginal	Ref			Ref			Ref			Ref		
Caesarean section	0.12	0.06–0.18	<.001	0.20	0.14–0.26	<.001	0.17	0.11–0.24	<.001	0.17	0.10–0.23	<.001
Birthweight	0.60	0.54–0.65	<.001	0.10	0.04–0.16	<.001	−0.27	−0.34 – −0.21	<.001	−0.08	−0.15–0.003	.04
Gestational age at birth, weeks	−0.06	−0.08 – −0.04	<.001	–	–	–	–	–	–	–	–	–
Gender												
Male	Ref			Ref			Ref					
Female	0.16	0.11–0.21	<.001	0.44	0.38–0.50	<.001	0.75	0.69–0.82	<.001	0.51	0.44–0.59	<.001
Breastfeeding at birth												
No				Ref			Ref			Ref		
Yes	−0.19	−0.24 – −0.14	<.001	−0.17	−0.23 – −0.12	<.001	−0.19	−0.24 – −0.13	<.001	−0.12	−0.21 – −0.03	.01
Welsh index of multiple deprivation												
Least deprived	Ref			Ref			Ref			Ref		
2	0.11	0.02–0.2	.02	0.13	0.04–0.23	.01	0.14	0.04–0.24	.01	0.13	0.02–0.23	.02
3	0.17	0.09–0.26	<.001	0.21	0.12–0.3	<.001	0.22	0.12–0.31	<.001	0.20	0.11–0.29	<.001
4	0.16	0.07–0.25	<.001	0.22	0.14–0.31	<.001	0.24	0.14–0.33	<.001	0.21	0.11–0.30	<.001
Most deprived	0.2	0.12–0.29	<.001	0.28	0.19–0.37	<.001	0.30	0.21–0.39	<.001	0.26	0.16–0.35	<.001
Urban/rural area of residence												
Urban	Ref			Ref			Ref			Ref		
Town and fringe	0.06	−0.01–0.12	.10	0.07	−0.01–0.14	.07	0.07	−0.01–0.14	.08	0.06	−0.01–0.14	.10
Village	0.06	−0.03–0.14	.20	0.06	−0.03–0.15	.21	0.05	−0.04–0.15	.27	0.06	−0.04–0.15	.23
Hamlet and isolated dwellings	−0.06	−0.18–0.06	.30	−0.06	−0.18–0.06	.34	−0.08	−0.21–0.05	.22	−0.06	−0.19–0.07	.40
Early-life weight (at model stage)	–	–	–	0.54	0.49–0.59	<.001	0.60	0.56–0.64	<.001	0.65	0.60–0.71	<.001
Child weight at 6 months	–	–	–	–	–	–	0.41	0.36–0.47	<.001	–	–	–
Breastfeeding at 8 weeks												
No	–	–	–	–	–	–	–	–	–	Ref		
Yes	–	–	–	–	–	–	–	–	–	−0.13	−0.23 – −0.03	.01
Transformations												
Maternal BMI (1)	(Maternal BMI)/100)^.5			(Maternal BMI)/100)			(Maternal BMI)/100)			(Maternal BMI)/100)		
Maternal BMI (2)	(Maternal BMI)/100)^2			(Maternal BMI)/100)^2			(Maternal BMI)/100)^2			(Maternal BMI)/100)^2		
AUC	0.67	0.67–0.68		0.72	0.71–0.73		0.78	0.77–0.79		0.79	0.78–0.80	
Calibration slope (standard error)	0.92 (0.04)			0.99 (0.01)			0.99 (0.01)			1.00 (0.01)		
Shrinkage factor	0.988			0.992			0.994			0.994		
Shrunken constant	−8.97	−8.99 – −8.94		−10.16	−10.19 – −10.15		−14.95	−14.98 – −14.93		−14.42	−14.44 – −14.39	

Only predictors significant at *P* < .157 were included in the prediction models. Categorical variables with at least one significant category have been included.

Discrimination (AUC) in the census subsample was 0.66 in the healthcare factors model, and 0.67 in the combined model with healthcare and census factors. Discrimination (AUC) in the early-life subsample was 0.67 in the birth model increasing to 0.79 in the model at ∼27months. Calibration slopes of all the models were close to 1 (0.92–1.00) indicating good calibration with slight overprediction in some models. Variables entered into the model mapped to the prenatal, antenatal, neonatal, and birth domain; demographic; transgenerational impact of parent health and health behaviours, socioeconomic; and neighbourhood, physical environment and health care systems (Table 4). Variables from all domains were retained in the model. All variables entered from the prenatal, antenatal, neonatal and birth and neighbourhood, physical environment, and health care systems were retained.

**Table 4.** Mapping of predictor variables to pre-conceptualized domains of early-life risk [18].

Domains	Variables	Entered	Retained in the census subsample	Retained in the early-life subsample
Prenatal, antenatal, neonatal and birth	Maternal age at pregnancy	Y	Y	Y
	Maternal BMI	Y	Y	Y
	Smoking during pregnancy	Y	Y	Y
	Parity	Y	Y	Y
	Mode of birth	Y	Y	Y
	Birthweight	Y	Y	Y
	Gestational age at birth	Y	Y	Y
	Breastfeeding at birth	Y	Y	Y
	Breastfeeding at 6-8 weeks	Y (early-life subsample only)		Y
Adverse childhood experiences	–	–	–	–
Child health including check-ups and screening	Child weight in early-life	Y (early-life subsample only)	–	Y
Developmental attributes	–	–	–	–
Child education and health literacy	–	–	–	–
Demographics	Gender	Y	Y	Y
	Ethnic group	Y	Y	Y
	Marital status	Y	Y	Y
	Main household language	Y (census subsample only)	N	–
Transgenerational impact of parent health and health behaviours	Parent disability affecting activities	Y (census subsample only)	N	N
	Parent health	Y (census subsample only)	N	N
	Unpaid carer	Y (census subsample only)	Y	Y
	Anaemia (mother)	Y	Y	N
	Asthma (mother)	Y	N	N
	Coronary heart disease (mother)	Y	N	
	Type 1 diabetes (mother)	Y	N	N
	Type 2 diabetes (mother)	Y	N	N
	Endometriosis (mother)	Y	N	N
	Epilepsy (mother)	Y	N	N
	Hyperthyroidism (mother)	Y	N	N
	Hypothyroidism (mother)	Y	N	N
	Irritable bowel disease (mother)	Y	N	N
	Learning disability (mother)	Y	N	N
	Polycystic ovarian syndrome (mother)	Y	N	
	Venous thromboembolism (mother)	Y	Y	N
	Mental health conditions (mother)	Y	N	
Socioeconomic	Maternal educational attainment	Y (census subsample only)	Y	–
	Number of cars in household	Y (census subsample only)	N	–
	Number of people per bedroom	Y (census subsample only)	N	–
Parental family factors and parental ability to care for a child	–	–	–	–
Neighbourhood, physical environment and health care systems	WIMD	Y	Y	Y
	Urban/rural area of mother’s residence	Y	Y	Y
Health behaviours and diet	–	–	–	–
Religion, spirituality and wider culture	–	–	–	–

Conditional on other variables in the model, maternal BMI was the strongest predictor in the model. Birthweight was also a strong predictor in the models at birth but less so when weight during early-life was included.

## Discussion

We developed models for predicting risk of childhood overweight/obesity at 4–5 years using linked healthcare and administrative data sources in Wales. Predictors retained from healthcare records were largely consistent with previously developed models in England [13]. However, additional census and area-level variables were retained though increase in model discrimination was marginal (0.66–0.67). In the early-life subsample, model discrimination increased from 0.67 at birth to 0.79 when incorporating child factors from early-life. Model discrimination at birth is poor but the maternal and birth factors are consistent across the stages which allows for early identification with more precise estimation as the child grows. Maternal BMI and birthweight are strong predictors and consistent across model stages. Factors from all domains that variables were mapped to were retained in the model.

The prediction model previously developed as part of the SLOPE study using healthcare data in Hampshire, England had a higher AUC using factors at birth (0.69) compared to these models developed using SAIL data (0.66 in census subsample using healthcare records only, 0.67 in early-life subsample) [13]. There were differences in variables available across the two datasets and how some variables were categorized. For example, breastfeeding at birth could not be included in model development in SLOPE due to high percentage of missingness but was retained in the SAIL models. Maternal smoking was available in both datasets but was categorized as never smoked, ex- and current smoker in SLOPE and non-smoker and smoker in SAIL so the non-smoker category could have included both never- and ex-smokers. Partnership status in SLOPE was a binary (yes/no) variable but had more detail in SAIL regarding whether mothers with a partner were living with the partner or not. Differences in included factors, definitions of factors and population characteristics can affect predictive performance [34] which may explain the lower AUC in the SAIL models.

Common predictors across the birth model in SLOPE and both SAIL subsamples were maternal age, maternal BMI, maternal smoking, ethnicity, parity, partnership status, birthweight, gestational age at birth and gender. Maternal educational attainment, intake of folic acid supplements and English as first language were available in SLOPE and retained as predictors in the model. Maternal educational attainment and English/Welsh as household first language was available in the Census data but only maternal education attainment was retained in the SAIL models.

Other variables retained in the SAIL model using the census subsample were if there were unpaid carers in the household and whether carer was working, WIMD, mother’s area type of residence at birth and maternal pre-existing health conditions (anaemia, venous thromboembolism). This demonstrates that the factors available in healthcare records such as maternal age, BMI, ethnicity, smoking status, parity and birthweight which are established childhood obesity risk factors are strong predictors.

In the SLOPE models, we found that discrimination improved when we added child weight at around 1 year (0.78) and 2 years (0.83), which are the points with statutory healthcare checks in England. These occur at slightly different ages in Wales—6, 15, and 27 months–and the AUC for models at these stages using the early-life subsample in SAIL followed a similar pattern (0.72 at 6, 0.78 at 15, and 0.79 at 27 months). Consistency of factors across the model stages means that high-risk groups could be identified early with more precise risk estimation as child grows.

The marginal increase in model discrimination on adding census factors implies that healthcare factors are more important for predicting risk of childhood overweight/obesity. This is in line with the literature with existing prediction models for overweight/obesity commonly including factors such as maternal BMI and birthweight, and health related behaviours such as maternal smoking during pregnancy [13, 35, 36]. Some models also include demographic data such as partnership status, maternal educational attainment or employment status, either from healthcare records [13] or using cohort data [28]. Household and area-level factors are less considered despite being wider determinants that contribute to risk which may be due to lack of recording in healthcare records or cohorts around early-life. Factors available in healthcare records usually include some measure of socioeconomic status and it is likely that socioeconomic status variables are correlated so adequate prediction may be achieved using the variables available in healthcare records.

Previous research has shown that early-life factors are associated with long-term health outcomes [37] and several early-life domains were found to be predictors of health outcomes in adulthood [38, 39]. Factors from all available domains were retained in the model in this analysis with all factors from the prenatal, antenatal, neonatal, and birth domain being retained. Prevention policy choices based on domains may help address wider determinants of health. For example, socioeconomic circumstances likely play an important role in the amount of resource available to parents towards achieving/maintaining a healthy weight, particularly with rising child poverty and food insecurity in England [40]. Risk stratification may help direct resources like healthy eating family vouchers or other financial or social support interventions towards those at highest risk.

A strength of this analysis is the population-based sample which enhances generalizability. We used robust statistical methods to develop the models (retained continuous variables as continuous by investigating variable transformations using multivariable fractional polynomials and calculated model shrinkage) and assess model discrimination. Routinely collected healthcare data was linked to administrative records which allowed to consider the household and area-level factors in predicting the risk of childhood overweight/obesity. Linking to other data sources in SAIL helped reduce missingness in some key factors but the high percentage of missing data led to some loss of information such as fewer classifications for maternal smoking during pregnancy. We additionally carried out multiple imputation of missing data enabling more robust analysis but requires caution in interpretation due to the number of imputations required. The use of BMI is a limitation as it does not distinguish between fat mass and fat-free mass, however age- and sex-adjusted BMI is recommended as a practical estimate of adiposity in children and young people to identify overweight and obesity.

Childhood obesity can act as a mediator on the pathway to multiple long-term conditions. Risk identification tools may be beneficial to target early prevention during antenatal and early-life care. The use of factors available in healthcare records makes the implementation of the risk prediction easier in healthcare settings. Risk could be quantified at birth as most maternal factors remained consistent across models with more precise estimation in early years.

## Supplementary Material

ckag051_Supplementary_Data

## Data Availability

Data may be obtained from a third party and are not publicly available. The data used in this study are available in the SAIL Databank at Swansea University, Swansea, UK. All proposals to use SAIL data are subject to review by an independent Information Governance Review Panel (IGRP). Before any data can be accessed, approval must be given by the IGRP. The IGRP carefully considers each project to ensure the proper and appropriate use of SAIL data. When approved, access is gained through a privacy-protecting trusted research environment and remote access system referred to as the SAIL Gateway. SAIL has established an application process to be followed by anyone who would like to access data via SAIL https://www.saildatabank.com/application-process. Key points There is currently no early identification system during pregnancy or early-life to detect those at high risk of childhood overweight/obesity. Wider demographic, socioeconomic and area-level data from administrative data could improve prediction of childhood obesity risk at school entrance age than using healthcare records alone. Additional area-level variables were retained though increase in model discrimination was marginal. Model discrimination at birth is poor but the maternal and birth factors are consistent across the stages which allows for early identification with more precise estimation as the child grows.

## Supplementary data

Conflicts of interest: R.K.O. is a member of the National Institute for Health and Care Excellence (NICE) Technology Appraisal Committee, member of the NICE Decision Support Unit (DSU)and associate member of the NICE Technical Support Unit (TSU). She has served as a paid consultant providing unrelated methodological advice to AstraZeneca, Cogentia Healthcare Ltd, Daiichi Sankyo, NICE, the Norwegian Institute of Public Health, Roche and Vifor Pharma. She reports teaching fees from the Association of British Pharmaceutical Industry (ABPI) and the University of Bristol. All other authors declare no further competing interests.
