# Supplementary figures and images for: Predicting childhood overweight and obesity at school entrance using healthcare, demographic, and socioeconomic data in Wales, UK
Source: Eur J Public Health. 2026 Apr 13;36(3):ckag051. doi: 10.1093/eurpub/ckag051 (PMC13075943; doi:10.1093/eurpub/ckag051)

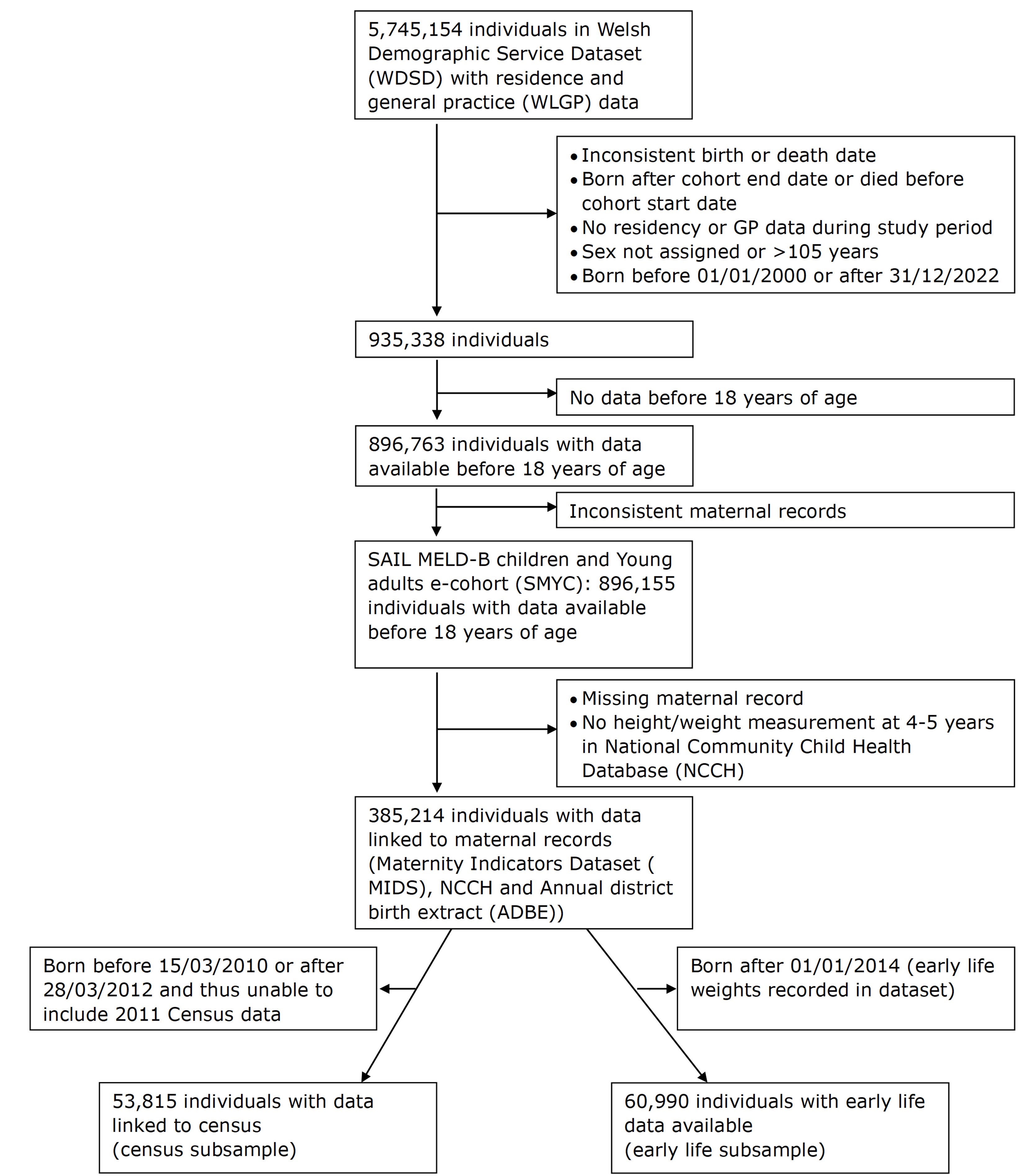

Supplement: ckag051_Supplementary_Data [file ckag051_supplementary_data.jpeg]
